# Dengue in Florida (USA)

**DOI:** 10.3390/insects5040991

**Published:** 2014-12-16

**Authors:** Jorge R. Rey

**Affiliations:** Florida Medical Entomology Laboratory, University of Florida-IFAS, 200 9th Street S.E., Vero Beach, FL 32962, USA; E-Mail: jrey@ufl.edu; Tel.: +1-772-778-7200 (ext. 136); Fax +1-772-7787205

**Keywords:** *Aedes aegypti*, *Aedes albopictus*, arbovirus, dengue, Florida, mosquito

## Abstract

Florida (USA), particularly the southern portion of the State, is in a precarious situation concerning arboviral diseases. The geographic location, climate, lifestyle, and the volume of travel and commerce are all conducive to arbovirus transmission. During the last decades, imported dengue cases have been regularly recorded in Florida, and the recent re-emergence of dengue as a major public health concern in the Americas has been accompanied by a steady increase in the number of imported cases. In 2009, there were 28 cases of locally transmitted dengue in Key West, and in 2010, 65 cases were reported. Local transmission was also reported in Martin County in 2013 (29 cases), and isolated locally transmitted cases were also reported from other counties in the last five years. Dengue control and prevention in the future will require close cooperation between mosquito control and public health agencies, citizens, community and government agencies, and medical professionals to reduce populations of the vectors and to condition citizens and visitors to take personal protection measures that minimize bites by infected mosquitoes.

## 1. Introduction

Dengue is a viral disease transmitted by mosquitoes that has caused serious public health problems in large parts of the world, particularly in urban and suburban areas of tropical and subtropical regions. Dengue is the most important arboviral disease on the planet, with an estimated 390 million infections yearly [1], more than 250000–500000 cases of the potentially fatal dengue hemorrhagic fever, and 25000 deaths per year [2]. The dengue virus (DENV) belongs to the family Flaviviridae and has five distinct serotypes (DENV-1, 2, 3, 4, 5). DENV-5 was recently discovered and has not been thoroughly investigated but transmission to humans in a sylvan cycle in Malaysia has been reported [2,3]. Infection by one serotype only provides partial and transitory immunity to infection with other serotypes. In fact, co-infection or sequential infection with different serotypes increases the risk of developing the more severe form of the disease called dengue hemorrhagic fever [4,5,6,7].

Even though dengue was historically considered mainly a zoonotic disease transmitted by forest mosquitoes to Old World primates [8], the disease has recently emerged as an urban malady maintained in most endemic areas by contact between mosquitoes, particularly *Aedes aegypti* (L.) and *Aedes albopictus* (Skuse), and human hosts. These mosquitoes occur in containers capable of holding water, both natural (tree holes, phytotelmata), and man-made (discarded tires, planters, buckets, *etc.*). Below we briefly review the occurrence of dengue in Florida, with emphasis on the more recent outbreaks in Monroe and Martin counties. Hribar [9] presents more details and insights on early dengue outbreaks in the State.

Although so far only *Ae. aegypti* has been implicated in dengue transmission in Florida, *Ae. albopictus* is also a capable host and has been known to transmit dengue in various locations including Brasil [10], Mexico [11], Hawaii [12], Macao [13] and other places [14]. The recent range expansion of this species in North America has been accompanied by decreases or complete local elimination of *Ae. aegypti* [15], but both species co-exist in many areas, and *Ae. aegypti* is re-appearing in some regions from where it was displaced. *Aedes aegypti* is usually more closely associated with urban habitats than *Ae. albopictus* and bites humans much more frequently than *Ae. albopictus*, [16,17]. Although several studies indicate that dengue infection rates in *Ae. albopictus* can be higher than in *Ae. aegypti*, [17,18,19], the closer association of *Ae. aegypti* with humans makes this species the better vector of dengue in our region, and the principal vector worldwide [11,17]. The role of *Ae. albopictus* in dengue transmission is still important as it can transmit the virus in areas where *Ae. aegypti* is not common, its interactions with the latter species can affect the dynamics of dengue transmission for both species, and its population dynamics can influence local disease prevention and vector control strategies. Ecological conditions of the immature stages of both species need to be taken into account when estimating vector capacity of local populations as these can exert significant influence on local disease transmission potential.

## 2. Dengue in the U.S.

Since Dr. Benjamin Rush described a disease resembling dengue from Philadelphia in 1780 [20], there have been recurring dengue outbreaks in the United States, particularly during the 19th and first half of the 20th century. In 1827–1828 disease outbreaks attributed to dengue took place in U.S. port cities such as New Orleans, Pensacola, Savannah, and Charleston. Well-known outbreaks occurred in New Orleans, Mobile, Charleston, Augusta and Savannah (1850); New Orleans (1873); Austin (1885), Galveston (1897, 1918, 1922), Louisiana (1922), and throughout Florida (1922, 1934), and Texas (1922, 1941). In many of these, over half of the population of the affected cities became ill with the disease.

Mosquito eradication programs undertaken after World War II, and presumably, lifestyle changes such as increased use of air conditioning, effectively interrupted the transmission of the virus to humans in the Continental U.S., but regular outbreaks have occurred in Puerto Rico and the U.S. Virgin islands during the 20th century. There was an outbreak in Hawaii in 2001, which represented the first appearance of the virus there since 1944. There were 25 cases in Brownsville, Texas, in 2005, 90 cases in Key West between 2009 and 2010, and 23 cases in Martin County, Florida in 2013 [8,9,20,21].

## 3. Dengue in Florida

Early reports of dengue in the State include those from the Dry Tortugas in 1903, and Miami in 1904 and 1908 [9]. More widespread outbreaks that affected close to 12,000 people occurred in 1905 and included Key West, Miami, Tampa, Jacksonville, and probably other locations. Outbreaks were reported from Hillsborough and Monroe counties in 1907, and in 1921–1922, the widespread dengue outbreak that affected Texas, Louisiana, Georgia, and Florida resulted in almost 83,000 cases and 69 deaths [22]. In 1934, another dengue epidemic affected a large portion of the State including Miami, Tampa and numerous other cities and populated areas and produced more than 1600 reported cases. At the time, it was estimated that more than 10% of the population of Dade County and that as many as 15,000 people in the city of Miami (pop. 135,000) were infected with the virus [9,23]. Locally transmitted dengue was also reported from Texas in 1986 and 1995.

## 4. Imported Dengue

**Figure 1:**
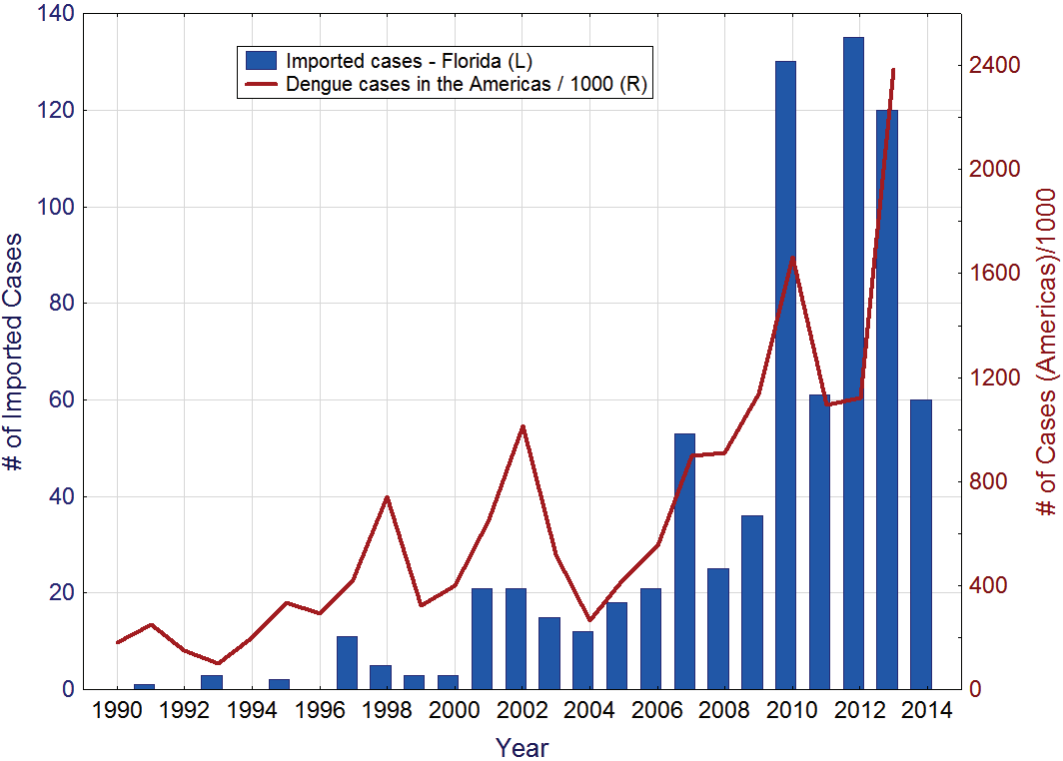
Imported dengue cases in Florida (left), and reported dengue cases in the Americas (right). Source: Pan American Health Organization [24].

During the last decades, imported dengue cases have been regularly recorded in Florida. The reemergence of dengue as a major public health concern in the Americas has been accompanied by a steady increase in the number of imported dengue cases in the state (Figure 1). Other contributing factors probably include a significant increase in travel, both by Florida residents to dengue endemic areas and by residents of those areas to Florida, and heightened vigilance and improved diagnostic protocols resulting in more cases being reported.

Multiple dengue serotypes are regularly imported into the State, from all corners of the globe. For example during 2013, 120 dengue case were imported into Florida from Angola, Bangladesh, Barbados, Bolivia, Brazil, the Caribbean, Colombia, Costa Rica, Cuba, Dominica, Dominican Republic, Haiti, Honduras, India, Indonesia, Jamaica, Mexico, Nicaragua, Nigeria, Panama, Philippines, Puerto Rico, Saint Martin, Trinidad and Tobago, Venezuela, and the U.S. Virgin Islands [25].

## 5. The Key West Outbreaks

**Figure 2:**
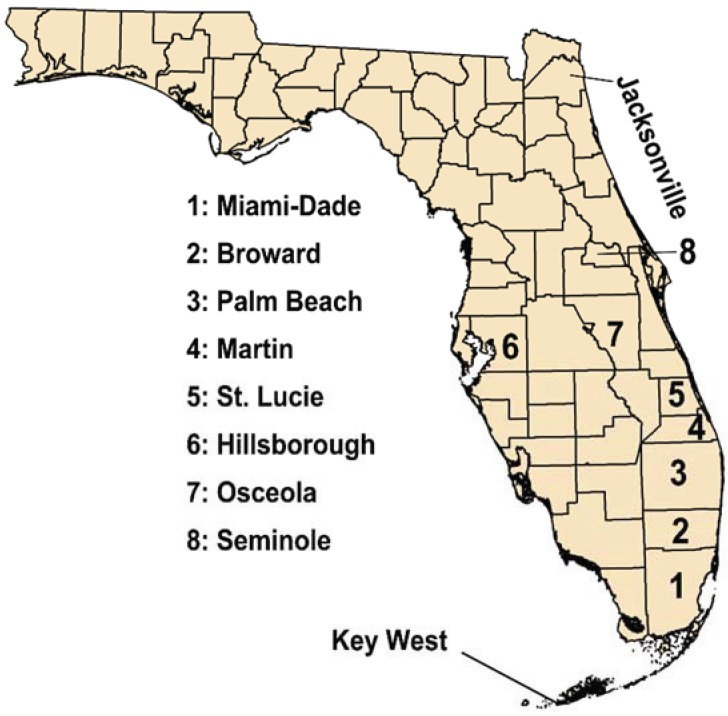
Map of Florida showing key locations discussed in the text (not to scale).

In 2009, a New York resident who had travelled to Key West (Monroe County, Figure 2) but nowhere else in the recent past was diagnosed with dengue fever and the diagnosis was confirmed by the US Centers for Disease Control (CDC) using PCR. This was the first case of autochthonous (locally acquired) dengue reported in Florida since the 1934 epidemic. Cases are considered locally acquired if the patient has no recent history of travel to areas where dengue transmission is occurring. Additional cases that developed dengue symptoms around August 25 and on September 9 were also confirmed as DENV-1 infections. Neither patient had travelled outside of Key West. Subsequently, more cases were reported until mid-October with a total of 28 locally acquired cases occurring.

In September 2009, the Florida Department of Health conducted serosurveys of residents in the Old Town section of the city where the reported cases were found, and discovered a 3%–6% infection rate among residents. Acute infections and some of the presumptive ones had antibodies against DENV-1, whereas 6% of the participants tested positive for past dengue infection with multiple serotypes [26,27]. Further analyses [28,29] indicate that the DENV-1 strains from Florida belong to a single genotype related to Central American strains. Positioning of the Key West strains within the derived phylogenetic trees indicate that sublineage formation is occurring in Monroe County, whereas other Florida strains clustered more closely with the original Central American strains [28].

**Figure 3:**
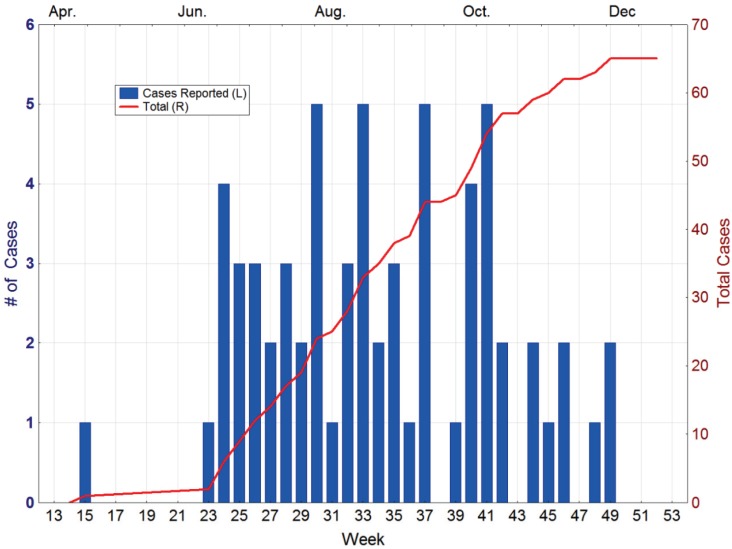
Dengue cases reported from Key West in 2010. Source Florida Department of Health [30].

In April of 2010, a 41-year-old man from Key West was diagnosed with dengue. Investigation by the Monroe County Health Department indicated that the disease was locally acquired, with onset in mid-March. No other cases were reported until mid-June, when another case was reported from Key West. From that point, new cases steadily appeared in the city until the end of November (Figure 3). In total, 65 cases of autochthonous dengue were associated with Key West during 2010, most of which (54) were local residents. Two other cases were reported from Florida that year, one each from Miami-Dade and Broward counties [30]. The Key West cases were all attributable to DENV-1 whereas the Miami-Dade case was DENV-2 and the one in Broward was DENV-3.

In subsequent years, sporadic locally-acquired dengue cases were reported throughout the State. In 2011, seven cases were reported, three from Miami-Dade, two from Palm Beach, and one each from Martin and Hillsborough counties. Only four cases occurred in Florida during 2012, two from Miami-Dade, one from Seminole and one from Osceola counties. Onset dates ranged from August to October [15].

## 6. Martin County 2013

In mid-August 2013, a dengue case was reported and later confirmed from Martin County in east-central Florida (Figure 2). Shortly thereafter, more cases were reported from Martin and neighboring St. Lucie County to the North. After further investigation, it was determined that the St. Lucie County cases were actually associated with Martin County. By the end of August, there were 11 cases associated with the small community of Rio (pop. ≈ 1000). By late September, when the last Martin County case was reported, there were a total of 22 cases of locally acquired dengue, from Rio and nearby Jensen Beach with onset from June through September. In mid-September, state and county health departments embarked in a serosurvey of Rio and Jensen Beach residents to estimate the extent of actual dengue infection in the area during the outbreak.

The September survey included 364 individuals from 256 randomly-selected dwellings in the Rio and Jensen Beach areas. Seven additional infections (1.9%) were identified, four of these asymptomatic, for a total of 29, all with DENV-1 [31]. It is unclear why the incidence rate in Martin Co. was lower than rates calculated from similar surveys conducted in Key West after the previous outbreaks (5%–6%), but it is likely that much less frequent exposure due to less travel and fewer visitors from infected areas played an important role. An additional dengue case was reported from Miami-Dade County in 2013. In 2014 five local cases have been reported as of October 4, all from Miami-Dade County [32].

## 7. Mosquito Control Response to Local Transmission

Because the mosquito vectors responsible for dengue transmission live in close proximity to people, and reproduce in large part in habitats provided by humans, control of these vectors requires a great deal of interaction between citizens and vector control agencies. This means that mosquito control agencies cannot quietly go about their business without too much public awareness of the actual transmission situation. It also means that no vector control program can be completely successful without considerable public cooperation. This situation can produce problems in areas like Key West, where the importance of the tourist industry to the local economy creates an automatic resistance to publicity of any factor, such as a disease outbreak, that could negatively impact tourism.

Citizen cooperation is essential in the control of dengue transmitting mosquitoes because only one or a few irresponsible property owners can produce enough mosquitoes to infest a whole neighborhood with potential disease vectors [33]. However, control is often seen as the sole responsibility of local and state mosquito control agencies, and lack of cooperation and even outright obstruction of mosquito control efforts by citizens and public officials are commonplace. This is a critical limitation to vector control efforts during dengue outbreaks.

Some arboviruses such as West Nile virus have intermediate hosts such as birds that can function as early warning monitors of active transmission in the area and trigger emergency vector control efforts before an actual human outbreak. DENV, on the other hand, has no intermediate hosts to perform this function so actual human cases are usually the first indication of active transmission in the region, further complicating the logistics of vector and disease control.

During the Key West outbreaks, the Florida Keys Mosquito Control District (FKMCD) acted aggressively, using all means at their disposal to reduce the populations of *Ae. aegypti*. This included increased surveillance, intensified larviciding and adulticiding, widespread use of lethal ovitraps, and site visits to affected neighborhoods to physically eliminate mosquito production sites. The FKMCD implemented their “Action to Break the Cycle of Dengue” (ABCD) campaign which included the use of public employees and volunteers to eliminate standing water sources (where mosquito immatures could develop) from public properties and rights-of-way. They also partnered with public utility and other agencies to transmit informational messages on dengue control in their customer correspondence such as bills, brochures, web sites, newsletters, and advertisements.

The FKMCD also embarked upon a direct public information campaign focused on residents, visitors, and business owners, explaining the importance of eliminating exposed water holding containers to reduce the abundance of dengue vectors, and of personal protection to minimize the probability of bites by remaining ones. Local reception to the latter efforts, however were lukewarm at best as once again fears of economic repercussions, in this case damage to the all-important tourist industry, became paramount. In many cases, citizens and business owners made no effort to mitigate mosquito production from their properties, some denied that disease transmission was taking place, and some went as far as accusing the FKMCD of exaggerating the situation in order to boost the agency’s funding [34].

In March of 2012, the FKMCD announced that it had partnered with the British firm Oxitec to release large numbers of genetically-modified *Ae. aegypti* males in Key West. The genetic modification produced by Oxitec causes matings with their mosquitoes to produce non-viable offspring. Release of large numbers of these mosquitoes is expected to reduce reproduction of natural populations, thereby decreasing their abundance and the probability of dengue transmission. As of this writing, FKMCD and Oxitec are awaiting FDA approval to initiate field trials of the technique.

Similarly, Martin County Mosquito Control and Health Departments responded quickly and forcefully to the discovery of dengue transmission in the county. Mosquito control was greatly intensified, and included increased larviciding, adulticiding, and source reduction. House-to-house inspections, treatments, and elimination of standing water sources were conducted in the Rio and Jensen Beach areas. These activities resulted in significant decreases in the proportion of dwellings infested with *Ae. aegypti* [31].

Special training courses for physicians developed by the CDC on the diagnosis and treatment of dengue were offered at two county hospitals. Vigorous public education campaigns were implemented by mosquito control and health agencies. Community response in Martin County was more positive than in Monroe as individuals and non-government organizations joined in the cleanup and information dissemination campaigns and garnered public support for the county’s vector control efforts [31].

## 8. Conclusions

Florida, particularly the southern portion of the State, is in a precarious situation concerning arbovirus transmission. The geographic location, climate, lifestyle, and the volume of travel and commerce, are all conducive to arbovirus transmission. In the last five years, Florida has experienced significant local transmission of emerging viruses such as dengue and chikungunya but have experienced only focal outbreaks of the disease. Although the probability of large scale epidemic transmission is lower than of localized outbreaks, events over the last five years demonstrate that the risks for either are not trivial. As with dengue, emergence of chikungunya in Latin America and the Caribbean was quickly followed by local transmission of the virus in Florida where this year 11 autochthonous cases and 260 imported cases of the disease have been reported as of October 4 [32].

Control and prevention of these viruses, transmitted by *Ae. aegypti* and the related *Ae. albopictus*, requires concerted efforts by mosquito control, public health agencies, citizens, community and government agencies, and medical professionals. Two major goals are paramount: (1) reduce populations of the vectors to interrupt (or prevent) transmission; and (2) condition citizens and visitors to take personal protection measures to minimize bites by infected mosquitoes during known or potential disease transmission episodes.

The first should involve an integrated vector management approach by mosquito control agencies that can include source reduction, adulticiding, larviciding, biological control, and other mosquito abatement techniques such as the use of lethal ovitraps, and sterile mosquito releases. It also must include a significant commitment by property owners to eliminate water-holding containers from their premises and an effective information campaign to buttress the critical need for continued vigilance and source reduction among both public and private sectors. The second will require education of citizens and visitors on realistic steps that can be taken by individuals to minimize their risk of infection. Both goals depend upon affecting a change in the prevalent public attitude that mosquito control and disease prevention is solely the responsibility of government agencies to one where individuals assume part of the responsibility for vector abatement and personal protection.

Disease outbreaks of any kind have the potential to cause catastrophic losses to a state where tourism represents a large part of the economy. The costs of treatment, long-term care, loss productivity, and other associated expenses can add significantly to the economic toll. Dengue and chikungunya are not the only mosquito transmitted diseases with potential to affect the state. Florida is also vulnerable to West Nile; eastern equine, St. Louis, and Venezuelan encephalitis; malaria; and many other mosquito transmitted diseases that are currently expanding from their historical geographic ranges [9]. Improved vigilance and cooperation between public agencies, civic organization, citizens, and visitors to the State are essential if we are to prevent major outbreaks of these emerging diseases in Florida.
